# Women’s perceptions of discussions about gestational weight gain with health care providers during pregnancy and postpartum: a qualitative study

**DOI:** 10.1186/s12884-017-1257-0

**Published:** 2017-03-24

**Authors:** Hara Nikolopoulos, Maria Mayan, Jessica MacIsaac, Terri Miller, Rhonda C. Bell

**Affiliations:** 1 Department of Agricultural, Food and Nutritional Sciences, University of Alberta, 4-126 Li Ka Shing Centre for Health Research Innovation, Edmonton, AB T6G 2E1 Canada; 2 University of Alberta, Community University Partnership, Facility of Extension, 2nd Floor, 2-281 Enterprise Square, 10230 Jasper Avenue, Edmonton, AB T5J 4P6 Canada; 3 Healthy Living, Population, Public and Aboriginal Health, Reproductive Health, Healthy Children and Families, Alberta Health Services, 10101 Southport Road SW cubicle #1740, Calgary, AB T2W 3N2 Canada

**Keywords:** Gestational weight gain, Pregnancy, Postpartum, Healthcare providers, Prenatal care, Antenatal care, Women’s perceptions

## Abstract

**Background:**

Maternal body weight is an indicator of the health of a mother and her developing fetus. Risks of poor maternal and fetal health issues increase when women gain too little or too much weight during pregnancy. A study of 600 women from Alberta, Canada, reported approximately 30, 46, 80, and 80% of underweight, healthy weight, overweight, and obese women, respectively, gained in excess of Health Canada gestational weight gain guidelines. Behavioural interventions during pregnancy have shown to be effective at supporting women achieve gestational weight gain (GWG) recommendations and return to their pre-pregnancy weight postpartum, yet few women are counseled about weight gain during pregnancy. A discrepancy exists between health care providers’ (HCP) reported counseling behaviours and women’s perceptions of counseling by HCPs; most HCPs report counseling women about GWG; conversely, most women report not receiving counseling about GWG. This study explored women’s experiences with GWG and their perceptions of discussions about GWG with HCPs during pregnancy and postpartum. This will help to identify gaps in service delivery and highlight areas for improvement that may better support women to achieve GWG recommendations leading to better health outcomes for women and children.

**Methods:**

Five focus groups (*n* = 26) were conducted with women up to 1 year postpartum across the five Alberta health zones. Focus groups were transcribed verbatim and analyzed using qualitative content analysis.

**Results:**

GWG is important to women, for their health and for the health of their baby. In-depth conversations with HCPs about GWG or weight loss do not occur; however, women want the opportunity to discuss weight gain/loss with HCPs. Women would like discussions about gestational weight gain/loss to become part of standard care and offered to all women.

**Conclusions:**

Women suggested that discussions about GWG should occur with all women, and that HCPs should initiate these discussions by asking women how they feel about discussing weight. Conversations should begin early on in pregnancy and continue through to the postpartum period. Interventions assessing discussions about GWG should be implemented and evaluated as this has been identified as a gap in prenatal service delivery.

## Background

Maternal body weight is an indicator of the health of a mother and her developing fetus. It is well known that the risks of poor maternal and fetal health issues increase when women gain either too little or too much weight during pregnancy [1]. While gaining too little weight is associated with low birth weight and preterm birth, excessive weight gain in pregnancy also contributes to increased rates of maternal and perinatal complications, illness and sometimes even death [2]. The combination of excess weight gain in pregnancy and poor diet quality, followed by less than recommended postpartum weight loss makes pregnancy a major risk factor for obesity and related chronic diseases (e.g. diabetes, certain cancers) in women in later life [3–5].

According to the 2006 Maternity Experiences Survey [6], only 47% of underweight women, 34% of healthy weight women, and 31% of overweight women achieved weights concordant with the 1999 Health Canada gestational weight gain (GWG) guidelines, while 26, 41, and 55% of underweight, healthy weight and overweight women, respectively, exceeded the guidelines. Similar statistics have been reported in several Western countries [7, 8]. Since then, Health Canada has adopted the updated 2009 Institute of Medicine GWG guidelines that reflect the WHO body mass index (BMI) categories and provide ranges of recommended weight gain for underweight, healthy weight, overweight, and obese women [9]. Recent studies indicate that although between 6 and 22% of women gain less weight than is recommended [10], the majority of women to gain in excess of recommended ranges. A study of 600 women living in Alberta, Canada, reported only 64% of underweight, 38% of healthy weight, 16% of overweight, and 14% of obese women met the 2010 Health Canada GWG guidelines, while approximately 30, 46, 80, and 80% of underweight, healthy weight, overweight, and obese women, respectively, gained in excess of the guidelines [11].

Behavioural interventions during pregnancy have shown to be effective at supporting women to achieve GWG recommendations [12] and return to their pre-pregnancy weight postpartum [13], yet few women report being counseled about weight gain during pregnancy [14] despite the fact that most healthcare providers’ (HCPs) reported counseling women about GWG [14–16]. It is possible that this discrepancy exists, in part, because little is known about how women perceive their interactions with HCPs when it comes to discussing GWG. For example, it is not known whether women would like to discuss GWG with their HCP, what women consider to be the most acceptable way to approach such discussion, at what point during the pregnancy women would like to discuss GWG, and the frequency that they want discussions to take place. This is important as many pregnant women have poor knowledge of GWG recommendations, and the consequences of inappropriate weight gain and of strategies to support appropriate GWG [17]. Furthermore, a review of the literature shows that behavioural interventions during pregnancy are primarily aimed at overweight and/or obese women but the importance of discussing GWG with women who begin pregnancy with a healthy BMI, between 18.5 and 24.9 kg/m^2^, has not been explored. There is a lack of published evidence of interventions that include healthy weight pregnant women, although gaining weight in excess of GWG recommendations is associated with postpartum weight retention [9] which is a strong predictor of overweight/obesity later on [12, 18].

The objective of this study was to gain an understanding of women’s experiences with GWG and their perceptions of discussions about GWG with HCPs during pregnancy and postpartum. Understanding women’s experiences with GWG will help to identify the supports women perceive are needed to help them achieve health pregnancy weight gain and postpartum weight loss. Findings will contribute to development of interventions aimed at improving interactions between HCP and women to support healthy pregnancy weight gain.

## Methods

### Study design

This study is one of several studies conducted in the ENRICH research program. ENRICH is a large multi-sector research partnership that uses an ecological framework, with the overall goal of improving maternal health by promoting optimal dietary intake and weight management in pregnancy and postpartum using innovative universal and selected strategies that meet the unique needs of women across Alberta. The present study is the qualitative portion of a study using mixed methods to explore knowledge, beliefs and practices of women in Alberta related to nutrition, physical activity and weight in pregnancy.

Using an exploratory qualitative methodology, focus groups were conducted to learn about women’s experiences with weight gain during pregnancy and weight loss during the postpartum period, and to learn about conversations women had with HCPs about GWG during and after pregnancy during routine visits. In exploratory studies, focus groups are useful in providing a variety of perspectives and encourage the exchange of opinions and ideas [19]. Focus groups are useful as, often, participants will build on what is said by others, contributing a wide breadth of experiences that may not otherwise have been explored.

This study was approved by the Health Research Ethics Board – Health Panel at the University of Alberta.

### Recruitment

Alberta Health Services provided permission and identified key staff to recruit women from Community/Public Health Centres in all five Alberta health zones (North, Edmonton, Central, Calgary, and, South). Women who were up to one year postpartum were eligible and were provided information about the study using information sheets, invitation postcards, Facebook posts, or during face-to-face interactions with staff at during “Well Child” immunization visits.. Women indicated their desire to participate in a focus group to Alberta Health Services staff and when ~5–8 women had done so the focus group was scheduled with the research group. This recruitment process helped to open the study to a wider group of potential participants than could have been recruited by the university-based study team.

### Data collection

Five focus groups were conducted (*n* = 26) with women across Alberta between July 2014 and September 2014. Focus group questions were semi-structured (including key probing questions), were developed through an iterative process among members of the ENRICH research team, and were pilot tested with a group of five women. All focus groups were conducted by two trained members of the ENRICH research team: the Program Manager (HN) and the Knowledge Translation Coordinator (JT). The Program Manager has formal training in qualitative methods and analyses and several years’ experience in this field [20, 21]. The Knowledge Translation Coordinator has experience in helping conduct focus groups in academic settings and within non-governmental organisations. Each focus group lasted approximately 90 min. Written informed consent was obtained from all participants at the beginning of each focus group. Participants were provided with a $25 grocery store gift card and baby bib in appreciation of their time. All participants completed a brief demographic questionnaire prior to beginning the focus group.

### Data analysis

Focus groups were digitally recorded, transcribed verbatim by a transcriptionist, and verified for accuracy by the Program Manager (HN) and the Knowledge Translation Coordinator (JT).

All focus group data were analyzed by the ENRICH Program Manager and the Knowledge Translation Coordinator using conventional content analysis: a process of inductively coding and categorizing data [22]. This approach is useful for exploratory studies as it allows for meaning to emerge from the data [23], rather than approaching analysis with preconceived theories, frameworks or ideas. Transcripts were analyzed separately then together by question to get an in-depth understanding of the data.

Transcripts were analyzed independently by a third reviewer external to the ENRICH team for coder reliability and validity of interpretations. Discrepancies were resolved through discussions about interpretation of findings until consensus was reached. Data saturation was achieved for each of the issues discussed.

## Results

The average age of participants was 30.4 ± 4.4 years, this was the first child for 80.8% of the women, average pre-pregnancy BMI was 24.4 kg/m^2^ ± 4.0, average GWG was 33.9 ± 18.9 lb., and type of HCP seen most often during pregnancy was: Obstetrician/Gynecologist (58.3%), Family Physician (37.5%), Midwife (4.2%). Age, BMI, GWG and type of HCP of this population are consistent with demographic data gathered from the nationally representative Canadian Maternity Experiences Survey (MES) [1].

Overall, the topics women discussed and the issues raised were consistent and reached satuation across the five focus groups. Data regarding women’s experiences with weight gain and weight loss, as well as their conversations had with HCPs are organized into three categories: 1) Women are concerned about gestational weight gain, 2) Communication with HCPs about GWG is lacking, and 3) Postpartum weight loss also matters.

### Women are concerned about gestational weight gain

Nearly all focus group participants identified that weight gain matters during pregnancy. Women reported that the amount of weight gained during pregnancy had implications for their health, the health of their pregnancy and the health of their baby. Weight gain was seen as a sign that the baby was in good health and that the pregnancy was moving along “on track”. Although weight gain was recognized as being important, it was also a source of concern.

Many women believed there was a "right amount" of weight to gain, specifically between 25 and 35 pounds. Women believed that gaining outside of this range (either more or less) may have negative implications for the health of their baby, which was a “big concern” for them.

*“…I was gaining way too much, way too fast and I was concerned about the health risk of that, to me and baby. And, yeah, I’m not so active, so…it coming off afterwards…I worried about that.”*

- *Participant E2*

Important to note, almost all women were confused about the range of weight gain (i.e., who it applies to and where this range comes from), and what the weight gain range meant in terms of rate and distribution of weight gain. Despite this, they believed this was the weight gain they should aim for, regardless of their pre-pregnancy BMI. Some women questioned the range. As one woman commented,

*“I’m just really curious on where the 25 to 35 pound range came from because there’s very, very few people that I’ve talked to who are anywhere near that 25 to 35 range. So I don’t know… Because I started to freak out about it, and then when everyone I talked to was like, oh no, I gained 50, 60, 70 pounds, I was like, okay, if everyone’s gaining that, then 50 is not too bad then. I’m okay.”*

- *Participant L*

As exemplified by the participant above, some participants’ expectations of pregnancy-related weight gain were based on past experiences and experiences of family and friends. For example, one participant thought she should gain what her mother had gained, which corresponded with what she had read in popular prenatal books so that is what she aimed for,

*“I thought I should gain what my mom gained. That’s where I got if from. And, it was right smack in the middle of what the books say, so I had that number in my head.”*

- *Participant E1*

When asked about how they were informed of a weight gain range, the majority of women reported accessing various resources, such as the books, *What to Expect When you’re Expecting* and *Healthy Parents, Healthy Children - Pregnancy and Birth* (Alberta Health Services); online websites, such as *Baby Centre, Fit Pregnancy, and What to Expect*; and searching “Dr. Google.” A few women reported this range was calculated by their HCP based on their pre-pregnancy BMI; however, most participants reported they did not receive information about an appropriate weight gain range from a HCP.

Participants reported varying levels of (dis)satisfaction with the amount of weight gained and their perceived ability to manage weight gain during pregnancy. For some women, gaining more weight than was recommended was frustrating and made them feel out of control. Some of these women stated that if they understood the implications exceeding recommendations could have on their baby, they would be more motivated to try to keep within GWG recommendations.

One participant noted that she “hated” the way she looked as she kept getting “thicker, thicker, thicker”. Some women struggled with changes in body shape (e.g., loss of muscle tone, feeling more “jiggly”). Others resigned themselves to the fact that weight gain was an inevitable part of pregnancy; they would try to lose the weight after the baby was born. Some women found it easy to cope with the amount of weight gained; however, for the few women who were satisfied or not concerned with their weight gain, they gained less than 30 pounds or less weight than they anticipated. They also believed they would be able to lose their weight quickly based on pregnancy experiences of family members and/or lifestyle behaviours (e.g. level of physical activity).

While women’s perceptions and experiences regarding weight gain varied, focus group discussions frequently centred on ways women tried to stay healthy and achieve healthy weights during pregnancy. Many women discussed monitoring and tracking their weight outside of doctors’ appointments on a frequent basis and almost all participants described modifications to their diet and/or amounts or types of physical activity. Modifications to diet included eliminating or reducing unhealthy foods, such as sweets, sodas and desserts, and increasing amounts of healthy foods, such as fresh fruits and vegetables. Conversely, some women noted that they “stopped being so strict” with their diet and would occasionally eat fast food because “now is the time to give in to your cravings”.

Changes to physical activity included both frequency and type of activity. The majority of women reported that they tried to walk more frequently and for longer periods of time. Others noted reducing the amount of physical activity due to high risk pregnancies (e.g., multiples, in vitro fertilization, extreme nausea), complications (e.g. sciatica, swelling), feeling tired or ill (mainly due to morning sickness), engaging in physical activities that were deemed not safe during pregnancy (e.g., heavy weight lifting), or due to family or social circumstances.

*“I didn’t want to screw anything up so I stopped running.”*

- *Participant E7*

Evident throughout the focus group discussions was stress, both good and bad, that pregnancy can place on a woman. Women talked about feeling stressed because they were “worried” they might do something wrong that could put the baby and pregnancy at risk. Women repeatedly reported experiencing feelings of guilt when they could not fully comply with GWG recommendations; make positive lifestyle changes; or when changes to their normal routine resulted in reducing healthy behaviours, such as exercising less or eating fewer healthy foods. Although women were motivated and aware of the importance of healthy lifestyle behaviours during pregnancy, this was not always simple or possible.

*“…it’s like, maybe earlier on I wasn’t eating the right things and I screwed this up and that’s why she’s not, you know, growing as much now. So, of course, you know, it kind of sets the wheels spinning… I’m trying to do the best I can here and…I don’t want to think I screwed this up…”*

- *Participant E6*

### Communication with HCPs about GWG is lacking

Women in all focus groups stated that communication with HCPs about GWG was lacking. An example of this was participants’ experiences of being weighed during prenatal appointments. Women described that they were weighed by nurses at nearly every prenatal appointment and that their weight was recorded in their chart but typically not disclosed or discussed with them. This lack of communication about weight gain was confusing, leading some participants to question if GWG was important to their HCPs.

*“I thought that maybe the obstetrician didn't really care about the weight I'm gaining because she didn't tell me too much…Every time, just go to the scale, she would look and tell me, ‘That's right’, every time. I don't know what's good or not.”*

- *Participant C3*

*“I got weighed at every appointment but no one ever – like, we never discussed whether I was, you know, gaining too much, too little, anything. Like, it was just never really brought up.”*

- *Participant N2*

Women who reported that conversations about GWG did occur explained that these conversations were neither timely nor positive. Several women commented that weight gain was discussed by HCPs only after they had gained too much weight or were “out of range”. One woman described that her weight was discussed by her physician, “only when I had done something bad” (i.e., gained too much weight in one month). This same participant expressed that she was “glad that they brought it [weight gain] up because that means they’re not ignoring it” but suggested that her doctor could have discussed concerns about weight in a “gentle way…to explore what could be happening”. Similarly, another woman recalled that her obstetrician “had this thing about weight gain; she made me feel kind of bad about it, that I had been gaining so much”.

Women that reported gaining too much total weight or gained weight too quickly felt that, often, HCPs made assumptions about their lifestyle behaviours resulting in feelings of frustration, humiliation, or distress. One woman, who had been running her entire pregnancy, commented that she was told by her doctor to “jump on the treadmill once in a while” because she had gained 40 pounds by approximately 30 weeks gestation and felt the physician never took the time to inquire about her level of physical activity.

*“I tipped the 40 pound scale, and that’s when she [the obstetrician] was like, ‘Whoa, whoa’, like, we hadn’t discussed it [GWG] at all up until that point [30 weeks] and then it was, okay, too much weight. But her recommendation was – and as far as I was… ’You should jump on the treadmill once in a while.’ And I ran until I was seven months pregnant, outside, because I like to run outside. And then I just – the weather wasn’t safe anymore. And so when she said, ‘Jump on a treadmill’, I was like, seriously? I was running the whole time.”*

- *Participant E6*

Again, another woman recalled gaining nine pounds in one month and the doctor “got a little cross with me”, telling her “you can’t be eating junk food”. This approach resulted in some participants experiencing negative emotions, such as guilt, blame, irresponsibility, and feeling out of control as more weight than was recommended was gained. These interactions with HCPs made women feel increasingly frustrated, commenting that the physicians’ approach to weight gain was “extreme” and “not helpful”, resulting in a lack of trust. As this participant described,

*“She [the obstetrician] should have asked me how I felt about my weight gain, not just told me how she felt about it.”*

- *Participant E2*

While HCPs communicated when too much weight was gained, many did not offer strategies to help or support women create plans to achieve recommendations, discuss with them how to adjust their expectations when things did not go as planned, or what to do for the remainder of their pregnancy once recommendations were exceeded.

Women wanted to be given the option to talk about weight gain and suggested that discussions about GWG should be done as early on in pregnancy as possible and as part of standard care. To do this, women recommended that HCPs could ease into conversations about weight by simply asking women if it is okay to talk about their weight. One woman stated that,

*“Not everyone likes to talk about their weight gain, so I guess they could ask if you like to talk about it.”*

- *Participant C2*

Women believe it is the responsibility of HCPs to broach the topic of weight gain and to provide accurate and timely information to women. Women want to discuss weight before exceeding recommendations, to be made aware of recommendations for total and rate of weight gain, their progress at every appointment; and they want regular feedback from their HCPs to assess if they are “on track”.

### Postpartum weight loss also matters

All women emphasized that it was important to return to their pre-pregnancy weight, with one woman stating “as fast as humanly possible”. Women commented that, after delivery, they felt that the focus of postpartum visits by public health nurses and physicians was on the baby; however, moms still matter and women want continued support and education from their HCPs. One woman stated that after the baby is born, “That’s the end of it. You’re sort of on your own now to deal with whatever”. Another woman reflected that HCPs do not discuss weight loss because they are more concerned about postpartum mental health. Another woman mentioned that women’s mental wellbeing and weight after pregnancy are often linked and, for that reason alone, physicians should be discussing postpartum weight loss.

Women could not recall ever discussing weight loss with their HCPs before or after giving birth, except for one participant whose midwife talked with her about, “losing the baby weight” while she was still pregnant. While the timing of this conversation was stressful at first, after her baby was born, she found it helpful to remember the midwife’s advice to focus on proper eating and not be overly focused about weight loss specifically. This participant wished she could continue discussions about weight loss at all of her postpartum visits; however, since seeing a family doctor, the issue had not been discussed.

The majority of women thought it would be best to have a weight loss discussion during the six week postnatal check-up; alternatively, some women thought it would be helpful to begin these discussions during pregnancy to have the opportunity to start thinking about it early on. Whether weight loss is discussed during or after pregnancy, all women agreed that they want to be given the option to have this discussion and that it should be a part of standard care.

*“No matter what size you are, I think every woman should have that [discussing postpartum weight loss] option. …if your healthcare provider sits there and says, here’s your options, it’s not like they’re telling you you’re heavy and you need to lose weight. They’re just saying that if you’re willing to or if you want to, here you go. And if they’re doing it to every woman, then every woman’s not going to feel cornered and saying, oh my gosh, you’re heavy. If it’s like, oh yeah, well, my healthcare provider asked me too, then they’re [women] going to be like, oh, okay, well maybe everybody’s getting asked. It’s not just me. It’s a woman thing. It’s every woman.”*

- *Participant V1*

## Discussion

Maternal body weight is simple to measure and widely used in many countries as a general indicator of maternal and fetal health throughout pregnancy [24]. Studies indicate that although maternal weight may be measured at most prenatal visits, between 45 and 80% of women exceed recommended amounts for total weight gain [6–8, 11]. This, in part, may reflect the fact that most women do not have regular, detailed conversations with their HCPs about appropriate GWG [14, 25].

Women reported that they had not had in-depth or regular discussions about GWG with their HCP, but that they would like to. They added further insight by indicating that they believed these conversations to be very important and they perceived HCPs as trusted and credible sources of information during pregnancy. Women believed there was a “right” amount of weight to gain to achieve a healthy pregnancy and that their weight in pregnancy was “more than just a number on a scale”. They also believed that the amount of weight gained was important for how they felt about their self-esteem, their health and the health of their baby. Lack of discussion about GWG with their HCP contributed to women’s confusion about what the right amount of weight to gain was and how to achieve it. It also contributed to women’s feelings of frustration and guilt when HCPs told them that they had gained an inappropriate amount. Our results support and extend the results from the USA [26] in which researchers conducted interviews with 24 overweight and obese women and found that discussions about GWG with HCPs did not occur but that women would like to have these discussions. Focus groups with pregnant women in France also noted that women look to their HCP for direction about health, eating and appropriate weight gain [27].

Women in our study stated that they wanted an opportunity for HCPs to learn about a woman’s lifestyle, health behaviours, beliefs and level of motivation so that HCPs could assist them in targeted and supportive ways.

Specifically, women wished:

- ➢ Discussions to begin early in pregnancy and occur regularly throughout pregnancy;
- ➢ To learn about the weight gain goals based on their pre-pregnancy BMI, including the trajectory of weight gain (i.e., approximate timing and distribution of weight gain);
- ➢ Regular and constructive updates about GWG to understand how their pregnancy is progressing;
- ➢ Specific advice and guidance about how to achieve weight gain goals and healthy pregnancies;
- ➢ Tailored advice about nutrition and physical activity; and
- ➢ HCP insights into anticipated changes to daily routines and lifestyles. Women in our study identified a large gap in prenatal care since only one woman reported that her HCP discussed postpartum weight loss. Women expressed great interest in wanting to discuss realistic weight loss targets (i.e., amounts and rates of weight loss) along with possible nutrition and physical activity strategies that would be safe and effective. Women suggested that they would welcome discussions about maternal postnatal weight and lifestyle during pregnancy and beginning at approximately 6 weeks postpartum. Relatively few studies have examined approaches to improve postpartum weight loss, although promising results on successful approaches using dietary interventions have recently been reported in Sweden [28–31]. New studies in the USA may shed additional light on how to positively impact on postpartum weight loss in a North American context [32].

Studies report that HCPs may avoid discussing GWG as this may be perceived to be a sensitive topic for women [33, 34]. Results from all of our focus groups were very consistent, with women stating that weight gain in pregnancy is expected and that they perceived weight gain to be an appropriate topic to discuss. Women felt they would be most open to discussions that began with HCPs asking women if they want to discuss weight gain/loss, and that discussions should be free of assumptions and judgment. Women stated that one way to approach GWG conversations would be for HCPs to ask all women about weight gain/loss, rather than restricting such discussions to women with a pre-pregnancy BMI in the overweight or obese category. Including regular discussions about GWG as part of standard practice with all women may contribute to improving women’s perceptions of these conversations. Our findings closely resemble those from [35] whose study was conducted with pregnant and postpartum women from the Boston area. The similarities between these 2 studies suggests that women with a low-risk pregnancy in the USA and Canada may face some similar issues related to GWG support and that they also identify some similar solutions.

### Limitations

This was an exploratory study of women’s experiences and their perceptions of discussions about GWG with HCPs during pregnancy and postpartum, and as such, only women’s perspectives are included. Women self-selected to participate in this study and, therefore, may be more motivated to share either positive or negative experiences than those who did not participate. However, we found that participants genuinely wanted to talk about their positive and challenging experiences with pregnancy. Most participants in this study were having their first child and it is possible that women’s perceptions and their needs for support may change in subsequent pregnancies. It was beyond the scope of this study to explore the extent to which women from different pre-pregnancy BMI categories or who experience different amounts or rates of GWG may prefer different types or amounts of support from their HCP, although this could be the focus of additional study.

Results from this study are most applicable in countries where physicians provide primary care to women during pregnancy, and where pregnant women are routinely weighed during prenatal visits. The need for enhancing the relationships between patients and their HCP has been identified in many areas of health care, including prenatal care [36–38]. Finally, although some focus groups were conducted with a small number of participants, findings were similar across the groups and saturation was reached.

## Conclusions

Women would like to have more regular and detailed discussions about GWG and postpartum weight loss with HCPs than occurred. Women in our study wanted discussions to be an opportunity for HCPs to learn about their lifestyle, health behaviours, beliefs and level of motivation so that HCPs could assist them in targeted and supportive ways.

This identifies an opportunity to improve prenatal care and could lead to better health outcomes for both women and children. Exceeding weight gain recommendations is a concern among pregnant women and increases the likelihood of retaining excess weight which can lead to chronic diseases in later life [39–41]. Women suggested that discussions about GWG should occur with all women, conversations should begin early in pregnancy as it is difficult to manage weight during pregnancy once weight gain recommendations and/or recommended rates of weight gain have been exceeded, and should continue through to the postpartum period. Interventions to promote healthy, supportive, patient-centred discussions about GWG should be implemented and evaluated to support improvements in perinatal health and practice.

## Abbreviations

BMI: Body mass index
GWG: Gestational weight gain
HCPs: Healthcare providers
